# Impact of Electronic Health Records on Long-Term Care Facilities: Systematic Review

**DOI:** 10.2196/medinform.7958

**Published:** 2017-09-29

**Authors:** Clemens Scott Kruse, Michael Mileski, Alekhya Ganta Vijaykumar, Sneha Vishnampet Viswanathan, Ujwala Suskandla, Yazhini Chidambaram

**Affiliations:** ^1^ College of Health Professions School of Health Administration Texas State University San Marcos, TX United States

**Keywords:** electronic health record, long-term care, benefits, patient outcome, quality, nursing home, electronic medical record

## Abstract

**Background:**

Long-term care (LTC) facilities are an important part of the health care industry, providing care to the fastest-growing group of the population. However, the adoption of electronic health records (EHRs) in LTC facilities lags behind other areas of the health care industry. One of the reasons for the lack of widespread adoption in the United States is that LTC facilities are not eligible for incentives under the Meaningful Use program. Implementation of an EHR system in an LTC facility can potentially enhance the quality of care, provided it is appropriately implemented, used, and maintained. Unfortunately, the lag in adoption of the EHR in LTC creates a paucity of literature on the benefits of EHR implementation in LTC facilities.

**Objective:**

The objective of this systematic review was to identify the potential benefits of implementing an EHR system in LTC facilities. The study also aims to identify the common conditions and EHR features that received favorable remarks from providers and the discrepancies that needed improvement to build up momentum across LTC settings in adopting this technology.

**Methods:**

The authors conducted a systematic search of PubMed, Cumulative Index of Nursing and Allied Health (CINAHL), and MEDLINE databases. Papers were analyzed by multiple referees to filter out studies not germane to our research objective. A final sample of 28 papers was selected to be included in the systematic review.

**Results:**

Results of this systematic review conclude that EHRs show significant improvement in the management of documentation in LTC facilities and enhanced quality outcomes. Approximately 43% (12/28) of the papers reported a mixed impact of EHRs on the management of documentation, and 33% (9/28) of papers reported positive quality outcomes using EHRs. Surprisingly, very few papers demonstrated an impact on patient satisfaction, physician satisfaction, the length of stay, and productivity using EHRs.

**Conclusions:**

Overall, implementation of EHRs has been found to be effective in the few LTC facilities that have implemented them. Implementation of EHRs in LTC facilities caused improved management of clinical documentation that enabled better decision making.

## Introduction

### Background

While birth rates are falling, life expectancy is rising in many countries, and people are entering an age when they will most likely need care [1]. Seventy percent of older people live in low- or middle-income countries [1]. As age increases, so does the prevalence of chronic illness [2]. The new trend in societies today is smaller families and different residential patterns leading to a rising need for paid care [3]. Health care systems need to find innovative and sustainable ways to cope with these changing demographics accompanied by changes in familial social patterns. In most countries, a significant percentage of people in the older age group needing long-term care (LTC) services rely on services provided by unpaid caregivers [3]. Organization for Economic Cooperation and Development (OECD) estimates show that 80% of all older citizens in Austria and 82.2% in Spain are dependent on informal home care [3]. Approximately, 62.8% American men and women over the age of 65 years will need LTC by 2050, and so will the 39.8% Western Europeans in their respective countries [4,5]. This reflects an international issue. A study conducted by the US Department of Health and Human Services (HHS) showed that 4 out of every 10 people aged 65 years will be enrolled in a nursing home at some point in their lives, and roughly 10% of these will stay for 5 years or more [6,7].

Governments around the world have responded to the rising need for LTC at various echelons of care for a range of acute and emergent illness or disease. Western European countries are underfunding their LTC needs, relying on existing national systems to manage acute and emergent services, but their health systems are not prepared to care for the countries’ dependent population for long periods [8]. For instance, most countries in Western Europe have a mechanism in place to fund formal care (50%-75% provided in the community), whereas Northern and continental European countries have arrangements to partially fund informal care [5]. Germany mandates LTC insurance, a program called *Pflegeversicherung*, to fund for LTC with equal contribution between the insured and their employers [8]. With the rising need for LTC and changing consumer expectations, some LTC facilities have been seen to adopt electronic health records (EHRs), despite the lack of funding opportunities, but overall the level of adoption of EHRs in the United States and Europe is low [8,9]. The EHR can improve quality of care in LTC facilities through a reduction in medication-related errors, improved clinical documentation and decision making, and through the Health Information Exchange. The latter point, which involves the Health Information Exchange, is particularly applicable to LTC because of the number of transfers and medical handoffs that accompany care of the elderly. These benefits are realized by the patient, the provider, and the organization. The EHR can also be associated with key qualities of both efficiency and effectiveness through improved data analysis and audits, coding and links to billing, going green or storage expenses, and record retention and proper safeguarding [8].

The Centers for Medicare and Medicaid Services (CMS) defines an EHR as “an electronic version of a patient’s medical history, that is maintained by the provider over time, and may include all the key administrative clinical data relevant to that person’s care under a specific provider, including demographics, progress notes, problems, medications, vital signs, past medical history, immunizations, laboratory data and radiology reports” [10]. Another organization within the CMS added the following to the definition: “allow access to evidence-based tools that providers can use to make decisions about a patient’s care, as well as automate and streamline provider workflow” [11]. With the help of the EHR, providers can access care-related activities directly or indirectly through various interfaces such as evidence-based decision support, quality management, and outcomes reporting.

In the literature, the terms EHR and electronic medical record (EMR) are often used interchangeably. The CMS differentiates these two: the EMR is bound to one organization, and the EHR is compatible across organizational lines. Although we would prefer that all publications kept these distinct, we also realize that it is impractical. Some of the literature analyzed in this review refers to EMRs when they are really analyzing an EHR. In the interest of keeping the authors’ words intact, we will not differentiate between them in our analysis. We are evaluating works about EHRs, EMRs, and some stand-alone components of the EHR or EMR for the same purpose of this review.

LTC is *a continuum of medical and social services designed to support the needs of people living with chronic health problems that affect their ability to perform everyday activities* [12]. LTC is an umbrella term that spans a large range of services. LTC services include traditional acute-care medical services, social services, skilled nursing facilities, rehabilitation facilities, assisted living, and other housing-based services. The goals of LTC are much more complicated and considerably more difficult to measure than the goals of acute medical care for the nonelderly.

The EHR enables providers to deliver better medical care to patients because of the availability of complete and accurate information [13]. Previous empirical studies conducted in other health settings consistently support that EHR can assist health care providers to minimize errors, improve safety and quality, and decrease costs [14]. The results from these empirical studies have influenced hospitals and other health care settings to implement and adopt EHRs actively; whereas LTC facilities, especially licensed nursing homes, have been slower in adopting and implementing EHRs [15]. This slower adoption pace is because of the lack of significant literature supporting the view that EHR implementation improves quality and decreases cost in the long run [16]. With the growth of aging population and LTC facilities providing care to this fast-growing segment, it seems important for these facilities to implement and use the EHR system meaningfully. Presence of the EHR in LTC could help meet the diverse needs of the dependent population and enable enhanced quality of care and coordination.

### Objective

The purpose of this review was to address the knowledge gap and the lack of significant literature accounting for the relationship between an EHR and LTC facilities. Do existing EHR implementations in current LTC facilities have positive outcomes? Do the users of these systems have positive experiences or observations that have been shared? The hypotheses are as follows:

H_1_: There are positive experiences by users of existing EHR implementations in LTC facilities.

H_0_: There are no positive experiences of users of existing EHR implementations in LTC facilities.

## Methods

### Eligibility Criteria

Our methods followed a measurement tool for the assessment of multiple systematic reviews (AMSTAR) [17]. The format of the review follows the preferred reporting items for systematic reviews and meta-analyses (PRISMA) [18]. Papers were eligible for selection in this systematic review if they were published in the last 10 years in academic (peer-reviewed) journals, in English, and whose full-text was available. We chose 10 years because we thought that 10 years would be a sufficient amount of time to collect information on technology. We limited the search to peer-reviewed journals to ensure some element of quality to the papers we were analyzing. We made the decision not to include other systematic reviews.

### Information Sources

We queried three common research databases: MEDLINE (the Web-based component to the MEDical Literature Analysis and Retrieval System) by Web of Science, Cumulative Index of Nursing and Allied Health Literature (CINAHL), and PubMed. We used key terms from the US National Library of Medicine’s Medical Subject Headings (MeSH) separated by Boolean terms. Searches were conducted from April 21 to April 24, 2017. The reason we chose to query MEDLINE by Web of Science is because we received different outputs when we queried MEDLINE in PubMed. We do not have a reason for the disparity. MEDLINE by Web of Science gave us more papers to choose from.

### Search and Study Selection

Searches in each database were nearly identical: (EHR OR EMR OR “electronic health record” OR “electronic medical record”) AND (“long term care” OR “long-term care” OR “nursing home”) AND (outcome OR impact OR effect) NOT “patient portal” NOT “health information exchange.” Due to the differences in indexing methods between the databases, we had to slightly modify the search string and filters for each. An exact listing of the search strings and filters is provided in Multimedia Appendix 1. We screened for date of publication to begin in 2007 and go through the end of April 2017. In PubMed and Web of Science, we were also able to screen out reviews. In both CINAHL and PubMed, we excluded MEDLINE because it was being collected separately from Web of Science. These 28 papers were placed into an Excel (Microsoft Corp) spreadsheet shared among the reviewers. The duplicates were removed.

### Data Collection Process and Data Items

Reviewers agreed ahead of time what to look for in each abstract. We wanted to focus on papers that explained an experience within an LTC facility of an EHR or a major component of the EHR as defined by the CMS [8,11]. We also searched for papers that expressed the experience, positively or negatively, in terms of effectiveness, for example, outcomes and quality, and/or or efficiency or advantages in money saved or workflow [19]. The initial search resulted in 100 results. After removing duplicates and filtering, the remaining 28 abstracts were divided among the reviewers in a way that all were reviewed at least twice (overlapping sets), as outlined by AMSTAR [17]. Reviewers carefully read each abstract ensuring that our review objectives were being addressed. Independent notes were taken on a shared spreadsheet. Additionally, each reviewer examined the references of each paper to identify any salient papers that our search may have missed, which identified an additional nine papers to the review queue. Before a consensus meeting, the Excel spreadsheets of each reviewer were combined to show agreement or disagreement about whether or not the paper was germane to our objective. An initial kappa statistic was calculated at kappa=.79. Where there was disagreement, reviewers discussed what they observed and reached a consensus. One reviewer on the team served as the facilitator and made the final ruling after hearing the input. Through this process, an additional seven papers were removed from consideration because of lack of applicability to our topic. At the end of the consensus meeting, the final selection of papers was chosen for analysis (N=28).

Papers were assigned to reviewers in a way that each paper was read by at least two reviewers. Once again, reviewers recorded independent observations on their copy of the Excel spreadsheet, ensuring to capture the observations in terms of effectiveness, efficiency, or the negatives of the same. Reviewers were also asked to identify possible bias in each paper, loosely following the Cochrane Collaboration’s risk-of-bias tool [20]. All observations were combined into one spreadsheet for discussion in the second consensus meeting. Reviewers were also asked to record any overarching themes that seemed to serve as a common thread between papers, as well as any significant levels of bias that could have been present in each study. This practice is in accordance with thematic analysis [21]. During the second consensus meeting, reviewers discussed their notes and observations (results, possible themes, and potential bias). No papers were discarded because of bias.

### Summary Measures and Synthesis of Results

**Figure 1:**
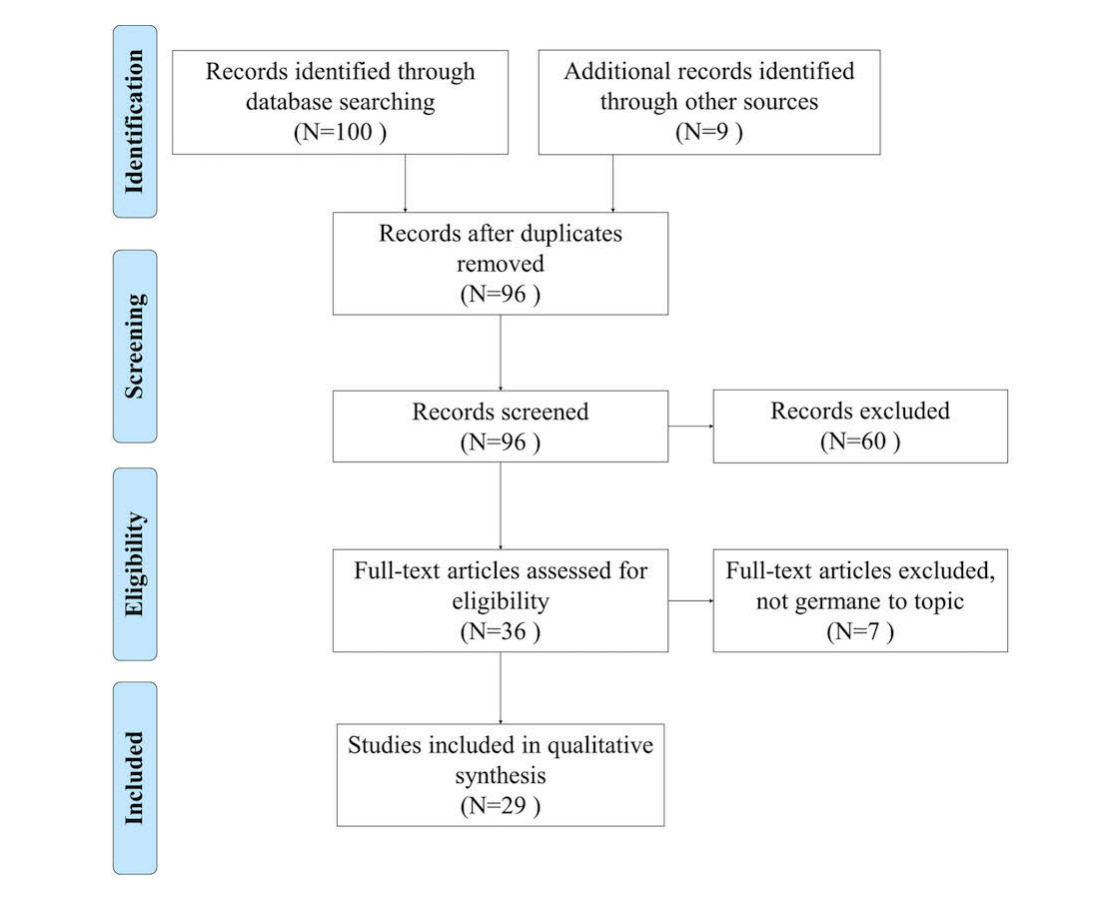
Paper selection process with inclusion and exclusion criteria.

The summary measure used in this analysis was the expression of experience with EHRs or a major stand-alone component of an EHR, in an LTC facility, expressed in terms of effectiveness or efficiency, and a frequency of occurrence of the themes identified by the reviewers. A table of observations was created, and an affinity matrix was created to illustrate potential trends. Figure 1 illustrates the selection process with the inclusion and exclusion criteria. This figure strictly follows the PRISMA standard.

## Results

### Study Selection and Characteristics

As illustrated in Figure 1, 100 papers entered the screening process, 13 duplicates were removed, 60 were screened out using our selection and exclusion criteria, 7 papers were removed because after reading their abstracts they did not seem to be germane to our objectives, and 9 additional papers were added from the references of those remaining. The final sample for analysis included 28 papers. Observations from each paper were summarized into our spreadsheet and from that spreadsheet we created a summary of the studies (Multimedia Appendix 2). Reviewers recorded observations of positive and negative experiences with the EHR in LTC, as well as any miscellaneous observations relevant for discussion.

### Additional Analysis

After the second consensus meeting, the overarching themes recorded by each reviewer were combined. We counted the number of times that a theme occurred in the literature and sorted by frequency of occurrence. These data were placed into an affinity matrix for further analysis (see Table 1). The total number of themes or attributes was 11, and the total number of occurrences was 44 [22-49].

The broad research criteria encouraged a thorough assessment of the implication of an EHR across various attributes: health outcomes, documentation, quality outcomes, length of stay, productivity, accessibility, medication safety, cost, patient satisfaction, nurses’ working time, physician satisfaction, and time consumption.

Among all the attributes, markedly 12 out of 28 papers addressed management of documentations, with approximately 27% of total occurrences of the attributes in the literature [24,27-33, 37,41,43,45,48]. Included in this attribute was data handling and the use of the EHR. Among these 12 papers, eight papers [24,29,32,34,37,41,43,48] showed positive impact, that is, improved management of documentation using EHRs, and five papers [27-30,45] portrayed negative impact of an EHR on documentation management, that is, it either increased documentation time and burden or showed no results post implementation.

**Table 1:** The frequency of occurrence of attributes to assess the impact of electronic health records (EHRs) in long-term care (LTC) facilities.

Attributes	Occurrences	Frequency (%)
Management of documentation	24,27-33,37,41,43,45,48	12 (27)
Quality outcomes	22,24,26,31,35,36,39,44,47	9 (20)
Health outcomes	24,28,31,41	4 (9)
Time consumption	26,27,36,49	4 (9)
Access to patient data	26,28,36,41	4 (9)
Physician satisfaction	31,37,38	3 (7)
Medication safety	42,44	2 (4)
Cost	23,41	2 (5)
Patient satisfaction	36,48	2 (5)
Productivity	46	1 (2)

Nine out of 28 papers (32%) reported positive quality outcomes, accounting for 21% of the occurrences [22,24,25,31,35,36, 39,44,47]. Four of 28 papers (14%) showed improved health outcomes using EHRs in aged care settings and nursing homes, accounting for 9% of occurrences [24,28,34,41]. Four out of 28 papers reported impact of EHRs on time consumption, accounting for 9% of occurrences [25,27,36,48], and surprisingly three out of these four papers showed negative impact, that is, time spent on all activities either remained unchanged post implementation or increased [25,28,36], and one paper reported reduced time consumption in creating electronic medical charts [48]. Four out of 28 papers (14%) demonstrated improved access to clinical information and patient data using EHRs, accounting for 9% of occurrences [25,28,36,41].

Notably, only three out of 28 papers (11%) reported greater physician satisfaction using EHRs, as it improved working environment and reduced errors [34,37,38]. Also, three attributes were mentioned only twice out of 28 papers: patient satisfaction [36,48], medication safety [42,44], and cost [23,41], each of which represent 5% of total occurrences of attributes in the literature. Furthermore, one attribute, which increased productivity of the settings, was reported only once out of 28 papers [46] after implementing EHRs, which represent only 2% of total occurrences.

Management of documentation was identified as a common theme in 13 papers. Studies documented that the time consumed for management of documents in EHR compared with paper-based records was significantly less [36,41,48]. Few papers also recorded that the management of documentation was more comprehensive, better in quality, and reduced human errors such as repetition and neglecting to medicate a resident [24,29,32,34,37,43,49]. One paper also emphasized the ease of documentation while using EHRs as compared with traditional paper-based documents [41].

Few papers mentioned that they could not observe much difference in the time consumed for documentation after implementing EHRs [28,33,45], and one among them mentioned that there was minimal difference initially which later increased the time taken 6 months after implementation but time taken increased 6 months after implementation [34]. One paper acknowledged the accuracy and comprehensibility of EHRs but also stated that these benefits were recorded in the first 6 months after implementing EHRs and were not sustained [37]. Reasons attributing to these unfavorable outcomes may, in part, be a result of the practice of documenting some information on paper and others on a computer. The lack of the staff’s experience with computer systems and the unavailability of required resources largely contribute to such outcomes. A more complex and in-depth understanding of the staff's perception, documentation workflow, and information needs along with sufficient resources and training might help in overcoming these results [30,33].

Quality outcome was the second most commonly observed theme. Many papers stated that EHRs directly improved the quality of care [25,34,36,44,47]. Another paper reported that the use of EHR improved interprofessional integration, thereby improving the quality of care.

Health outcome was another commonly identified theme. Four different papers showed significant improvement in health outcomes by reducing the occurrence of infections, high-risk pressure sores, neurolepsis, improving activities of daily living (ADL), range of motion, and timely medication [41,34]. One study particularly emphasized that the likelihood of neglecting to medicate a resident decreased but also noted that there were unintended incidents of neglect to medicate because of energy blackouts [24]. There was another study that mentioned that, when applied to delirium prevention strategies, EHRs failed to lower delirium rates among patients with hip fracture. Factors such as staff turnover, impact of organizational culture, personnel changes, and structure on the uptake of the delirium prevention strategies were the major factors that influenced the failure of this model. Furthermore, there were multiple challenges operating at different levels within the system [40].

The next most commonly occurring attribute was time consumption. In a few of the studies, the respondents have mentioned that EHR was time consuming because of reasons such as complexity in signing out of an EHR [25,36]. Another paper stated that there was no significant change in the proportion of time spent on activities and oral communication [27]. General physicians in a study had responded that the time taken to create electronic medication charts was much less compared with conventional charting [48]. Out of four papers that refer to time consumption, three state that there is no evidence of time saving as a result of using EHRs. This shocking observation calls for more research to address the time-consuming aspect of EHRs.

Access to patient data was another commonly occurring attribute [41,25,27,36]. Out of these papers, three mentioned that EHRs improve access to patient records by facilitating real-time availability and remote access [41,25,36]. One study stated that implementation of EHRs resulted in difficulty to access data [28].

Other common factors included cost, patient satisfaction, physician satisfaction, length of stay, and productivity. Studies mentioned that there was a marked increase in the cost incurred by facilities post implementation [41,35]. The authors recommend that further research should attempt to throw light on the factors contributing to increase in cost and evaluate ways by which the high upfront cost could be balanced with benefits in LTC facilities, as this would inspire more facilities to adopt EHRs.

## Discussion

### Summary of Evidence

EHRs are known to improve care coordination and health outcomes. Although LTC facilities have been slow to adopt EHR, they continue to be areas where the benefits of implementing EHR can be realized to its fullest potential. This review tries to identify the established outcomes in various LTC facilities that have adopted this technology. For this review, we analyzed papers, studies, and other summaries of experiences relating to our topic of interest. Management of documentation, quality outcome, and health outcomes were identified as the most common themes, which were identified in 60% of all papers reviewed.

There were both positive and negatives outcomes reported in this systematic review; however, the former was found in the literature more than the latter. Some reported a boost in productivity only after 23 months; others did not put a time frame on it—they just reported slower processes.

The LTC market has been slow to adopt health information technology, in general, and EHRs specifically. The paucity of data on the adoption of the EHR in LTC is similar to the private health care market in the United States before the major legislation in 2009. The adoption rates for EHRs in the United States greatly increased with incentives that helped to offset the steep adoption costs of the technology. Future research could determine the level at which the cost of investing in the EHR is equal or better than the cost of abstaining.

### Limitations

The researchers reviewed only those papers that were published between the years 2007 to 2017 and did not include the papers outside the period of study. We thought 10 years’ time was adequate, commensurate with other reviews. There is unavailability of data owing to the slow adoption of EHR in LTC settings. The systematic search process in the three primary databases yielded studies that predominantly focused on the United States’ LTC scenario rather than an international focus. Although selection bias and face validity are concerns, we mitigated these risks by following the AMSTAR standard and using more than one reviewer to opine on the inclusion or exclusion of papers used for analysis [17].

### Conclusions

Overall, implementation of EHRs has been found to be more effective than not in LTC facilities. Implementation of EHRs in LTC facilities caused improved management of clinical documentation that enabled better decision making. Negative experiences were observed in workflow and productivity, but it is unclear whether this was because of change management and the general disruption that a major information technology (IT) implementation can exert on the organization. The authors recommend improving the design of EHRs that address issues such as time spent on documentation and enhancing the usability for physicians and nurses. These improvements would address most of the negative experiences and may promote widespread adoption of this essential technology in LTC.

## Appendix

Multimedia Appendix 1 MEDLINE by Web of Science (Thompson Reuters).

Multimedia Appendix 2 Summary of analysis.

## Abbreviations

ADL: activities of daily living
AMSTAR: assessment of multiple systematic reviews
CINAHL: Cumulative Index of Nursing and Allied Health Literature
CMS: Centers for Medicare and Medicaid Services
HHS: US Department of Health and Human Services
EHR: electronic health records
EMR: electronic medical records
HIT: health information technology
LTC: long-term care
MeSH: medical subject headings
OECD: Organization for Economic Cooperation and Development
PRISMA: preferred reporting items for systematic reviews and meta-analyses
RAC: residential aged care
RACF: residential aged care facility
