# Supplementary material for: Impact of Electronic Health Records on Long-Term Care Facilities: Systematic Review
Source: JMIR Med Inform. 2017 Sep 29;5(3):e35. doi: 10.2196/medinform.7958 (PMC5640822; doi:10.2196/medinform.7958)
Supplement: Multimedia Appendix 1 [file medinform_v5i3e35_app1.pdf]

Search string: You searched for: ((((TOPIC: (EHR) OR (TOPIC: (EMR) OR MeSH HEADING:exp: (Electromagnetic Radiation) OR MeSH HEADING:exp: (Electronic Health Records) OR (MeSH HEADING:exp: ((Therapeutics)) AND MeSH HEADING:exp: ((Endoscopy, Gastrointestinal))))) OR TOPIC: ("electronic health record")) OR TOPIC: ("electronic medical record")) AND (((TOPIC: ("Long term care") OR MeSH HEADING:exp: (Long-Term Care)) OR TOPIC: ("Long-term care")) OR TOPIC: ("nursing home"))) AND TOPIC: ((((outcome) OR impact) OR effect) NOT "patient portal") NOT "Health information exchange")

Refined by: MeSH HEADINGS: (HUMANS) AND [excluding] PUBLICATION TYPES: (LETTER OR EDITORIAL OR REVIEW) AND PUBLICATION YEARS: (2014 OR 2009 OR 2015 OR 2016 OR 2017 OR 2010 OR 2012 OR 2008 OR 2013 OR 2007 OR 2011)

Timespan: All years (2007 – 2017). Indexes: MEDLINE.

(n=34). The filters used were humans, 2007-2017, exclude reviews, editorials, and letters,

CINAHL

(EHR OR EMR OR "electronic health record" OR "electronic medical record") AND ("Long term care" OR "Long-term care" OR "nursing home") AND (outcome OR impact OR effect) NOT “patient portal” NOT "Health information exchange“

(n=28)

Filters: English, humans, 2007-2017, academic journals, exclude reviews, editorials, letters.

PubMed

(("Environ Hist Rev"[Journal] OR "ehr"[All Fields]) OR ("Empir Musicol Rev"[Journal] OR "emr"[All Fields]) OR "electronic health record"[All Fields] OR "electronic medical record"[All Fields]) AND ("Long term care"[All Fields] OR "Long-term care"[All Fields] OR "nursing home"[All Fields]) AND (outcome[All Fields] OR ("Impact (Am Coll Physicians)"[Journal] OR "impact"[All Fields]) OR effect[All Fields]) NOT "patient portal"[All Fields] NOT "Health information exchange"[All Fields] AND ("loattrfull text"[sb] AND "2007/06/27"[PDat] : "2017/06/23"[PDat] AND "humans"[MeSH Terms] AND English[lang] AND (jsubsetaim[text] OR jsubsetn[text]))

Filters: full text, 10 years, English, core clinical journals, nursing journals,
