# Supplementary material for: Impact of Electronic Health Records on Long-Term Care Facilities: Systematic Review
Source: JMIR Med Inform. 2017 Sep 29;5(3):e35. doi: 10.2196/medinform.7958 (PMC5640822; doi:10.2196/medinform.7958)
Supplement: Multimedia Appendix 2 [file medinform_v5i3e35_app2.pdf]

Title	Findings Relevant to Review
The Impact of Electronic Health Records on Risk Management of Information Systems in Australian Residential Aged Care (RAC) Homes [22].<br>Original research, primary data collection.	**Effectiveness**: Australian RACs used EHRs in risk management and meeting information system accreditation standard. The extent of RAC homes utilizing EHR that met all accreditation measures was altogether higher than that of homes with paper records.
Meaningful Use of EHRs Among Hospitals Ineligible for Incentives Lags Behind That of Other Hospitals, 2009-13 [23].<br>Original research, secondary data collection.	Inconclusive efficiency: While costs slow adoption rates among LTC facilities, growth rates do not seem to be affected.
The impact of electronic medication administration records in a residential aged care home [24].<br>Original research, primary data collection.	**Effectiveness**: This study demonstrated advantages of presenting electronic medication administration records included enhancing nurses' consistence with documentation necessities, reducing the error of signing twice, decreasing the likelihood of neglect to medicate a resident, encouraging medical attendants to record the time of medication administration to a resident, and expanding documentation space.<br>**Ineffectiveness**: Deficient data about inhabitants, late expansion of another occupant's medicine profile in the records and nurses' neglecting to medicate an occupant because of energy blackout.
Electronic Health Records in Long-Term Care: Staff Perspectives [25].<br>Original research, primary data collection.	**Effectiveness**: This study described patterns of use and areas to improve with an EHR in a Midwest United States long-term, post-acute-care setting. There was also documentation for positive effect on quality.<br>**Efficiency**: The majority of respondents evaluated the EHR as easy to use, and greater efficiencies through communication with the care team.<br>**Inefficiencies**: Staff responses outline desired modifications to the software, including fixes to data fields for more accurate medication administration and accurate reports on bowel protocol follow-up. Signing out seemed unnecessarily time consuming.
Bringing care home: how telemonitoring can expand population health management beyond the hospital [26].<br>Report.	**Effectiveness**: EHR data supported predictive ability of discharge model, which in turn reduced the risk of readmission.

Title	Findings Relevant to Review
The effect of an electronic health record system on nursing staff time in a nursing home: a longitudinal cohort study [27].<br>Original research, primary data collection.	Inconclusive results for effectiveness and efficiency:<br>• No difference in time spent on activities before and after EHR implementation,<br>• no change in proportion of time spent on oral communication, except at 23 months' post-implementation,<br>• no major change was seen in documentation time, except at 23 months' post implementation time spent on all activities remained unchanged after introduction of her.
Unintended adverse consequences of introducing electronic health records in residential aged care homes [28].<br>Report	**Ineffectiveness**: This study showed negative results after introducing EHRs in residual aged care facilities like difficulty in access,<br>**Inefficiency**: Increased complexity in information management, increased documentation burden, reduction of communication and the risks of lacking care follow-up.
Description and comparison of quality of electronic versus paper-based resident admission forms in Australian aged care facilities [29].<br>Original research, secondary data collection.	**Effectiveness**: Results indicated that overall completeness and comprehensiveness rates of the admission forms were higher using electronic health records compared to paper-based records and enhanced quality of documentation in resident admission forms was recognized in the electronic documentation frameworks.
Caregivers' Time Utilization before and after the Introduction of an Electronic Nursing Documentation System in a Residential Aged Care Facility [30].<br>Original research, primary data collection.	**Inefficiency**: The amount of time spent by personal care givers on documentation increased.
Can Electronic Tools Help Improve Nursing Home quality [31].<br>Original research, primary data collection.	**Effectiveness**:<br>• Increased proportion of patients timely taking their doses<br>• Patients taking neuroleptics gradually decreased from 33 to 25 percent<br>• No change in internal controls

Title	Findings Relevant to Review
Aged Care IT in Australia – the Past, Present and Future [32].<br>Original research, primary data collection.	**Effectiveness**: The use of HIT in the LTC community gives ready access to specialty care services who look for patients that way. Efficiency: EHRs can facilitate the business end of aged care. The use of HIT in aged care forces legislation on accountability, security, privacy, and confidentiality. EHRs can also increase communication within a facility with lead to secondary effects of efficiency.
Does the introduction of an electronic nursing documentation system in a nursing home reduce time on documentation for the nursing staff [33].<br>Original research, primary data collection.	**Inefficiencies**: There was no significant difference in efficiency using an electronic nursing documentation system.
The benefits of introducing electronic health records in residential aged care facilities: A multiple case study [34].<br>Original research, primary data collection.	**Effectiveness**: Residential aged care facilities (RACFs) demonstrated improvements in quality of care.<br>Efficient: RACFs demonstrated improvements in data management, working environment and educational benefits.
Experiences with electronic health records: Early adopters on long-term care facilities [35].<br>Original research, primary data collection.	**Effectiveness**: implementation of evidence-based practices<br>**Efficiency**: operational improvements were achieved through increased access to resident information, cost avoidance, increased documentation accuracy.
The Use of Bedside Electronic Medical Record to Improve Quality of Care in Nursing Facilities: A qualitative analysis [36].<br>Original research, primary data collection.	**Effectiveness**: The results reported that the use of a bedside EMR in nursing homes improved quality of care, better communication with physicians, and easy access to clinical information.<br>**Inefficiency**: Staff reported EHRs to be time consuming.
The changes in caregivers' perceptions about the quality of information and benefits of nursing documentation associated with the introduction of an electronic documentation system in a nursing home [37].	**Effectiveness**: The caregivers perceived EHR as a provision of accurate, legible and complete information. They reported that there is a reduction in data repetition errors. These benefits were recorded during first 6 months after introducing EHR, but were not maintained at 18-21 months.

Title	Findings Relevant to Review
Original research, primary data collection.	
Advice for Decision Makers Based on an Electronic Health Record Evaluation at a Program for All-Inclusive care for the elder sites [38]. Original research, primary data collection.	**Inefficiency**: Results showed that where the EHR utility was complex to work with or did not assist the clinical workflow, clinicians did not adopt the EHR as expected.<br><br>Initially, clinicians were dissatisfied, but upon usage they showed increased satisfaction
Integrated care through disease-oriented clinical care pathways: experience from Japan's regional health planning initiatives [39]. Original research, primary data collection.	**Efficiency**: These ‘inter-provider critical paths’ ensure effective integration (hand-offs) between various providers and levels of care ranging among primary care practitioners, acute care hospitals, rehabilitation hospitals, long-term care facilities and home care.
A pragmatic study exploring the prevention of delirium among hospitalized older hip fracture patients: Applying evidence to routine clinical practice using decision support [40]. Original research, primary data collection.	**Ineffectiveness**: The electronic medical records when applied to delirium prevention strategies, failed to decrease delirium rates among the patients with hip fracture

22. Jiang T, Yu P, Hailey D, Ma J, Yang J. The Impact of Electronic Health Records on Risk Management of Information Systems in Australian Residential Aged Care Homes. *Journal of medical systems*. 2016 Sep 1;40(9):204.

23. Walker D, Mora A, Demosthenidy MM, Menachemi N, Diana ML. Meaningful Use Of EHRs Among Hospitals Ineligible For Incentives Lags Behind That Of Other Hospitals, 2009–13. *Health Affairs*. 2016 Mar 1;35(3):495-501.

24. Qian S, Yu P, Hailey DM. The impact of electronic medication administration records in a residential aged care home. *International Journal of Medical Informatics*. 2015 Nov 29;84(11):966-73.

25. Meehan R. Electronic Health Records in Long-Term Care Staff Perspectives. *Journal of Applied Gerontology*. 2015 Oct 12:0733464815608493. DOI: https://doi.org/10.1177/0733464815608493

26. Aguilar KM, Campbell RS, Fiester A, Simpson RL, Hertel C. Bringing care home: how telemonitoring can expand population health management beyond the hospital. *Nursing administration quarterly*. 2014 Apr 1;38(2):166-72.

27. Munyisia E, Yu P, Hailey D. The effect of an electronic health record system on nursing staff time in a nursing home: a longitudinal cohort study. *The Australasian medical journal*. 2014;7(7):285.

28. Yu P, Zhang Y, Gong Y, Zhang J. Unintended adverse consequences of introducing electronic health records in residential aged care homes. *International journal of medical informatics*. 2013 Sep 30;82(9):772-88.

29. Wang N, Yu P, Hailey D. Description and comparison of quality of electronic versus paper-based resident admission forms in Australian aged care facilities. *International journal of medical informatics*. 2013 May 31;82(5):313-24.

30. Munyisia EN, Yu P, Hailey D. Caregivers' time utilization before and after the introduction of an electronic nursing documentation system in a residential aged care facility. *Methods Inf Med*. 2013 Jan 1;52(5):403-10.

31. Krüger K, Strand L, Geitung JT, Eide GE, Grimsmo A. Can electronic tools help improve nursing home quality?. *ISRN nursing*. 2011 Oct 10;2011.

32. Yu P. Aged care IT in Australia-the past, present and future. *Electronic journal of health informatics*. 2012;7(2):1-3. ISSN:1446-4381

33. Munyisia EN, Yu P, Hailey D. Does the introduction of an electronic nursing documentation system in a nursing home reduce time on documentation for the nursing staff?. *International journal of medical informatics*. 2011 Nov 30;80(11):782-92.

34. Zhang Y, Yu P, Shen J. The benefits of introducing electronic health records in residential aged care facilities: a multiple case study. *International journal of medical informatics*. 2012 Oct 31;81(10):690-704.

35. Cherry BJ, Ford EW, Peterson LT. Experiences with electronic health records: Early adopters in long-term care facilities. *Health care management review*. 2011 Jul 1;36(3):265-74.

36. Rantz MJ, Alexander G, Galambos C, Flesner MK, Vogelsmeier A, Hicks L, Scott-Cawiezell J, Zwygart-Stauffacher M, Greenwald L. The use of bedside electronic medical record to improve quality of care in nursing facilities: a qualitative analysis. *CIN: Computers, Informatics, Nursing*. 2011 Mar 1;29(3):149-56.

37. Munyisia EN, Yu P, Hailey D. The changes in caregivers' perceptions about the quality of information and benefits of nursing documentation associated with the introduction of an

electronic documentation system in a nursing home. *International journal of medical informatics*. 2011 Feb 28;80(2):116-26.

38. Sockolow PS, Weiner JP, Bowles KH, Abbott P, Lehmann HP. Advice for decision makers based on an electronic health record evaluation at a Program for All-inclusive Care for Elders site. *Appl Clin Inf*. 2011 Jan 1;2(1):18-38.

39. Okamoto E, Miyamoto M, Hara K, Yoshida J, Muto M, Hirai A, Tatsumi H, Mizuno M, Nagata H, Yamakata D, Tanaka H. Integrated care through disease-oriented clinical care pathways: experience from Japan's regional health planning initiatives. *International Journal of Integrated Care [serial online]*. 2011 Jan 1.

40. Holroyd-Leduc JM, Abelseth GA, Khandwala F, Silvius JL, Hogan DB, Schmaltz HN, Frank CB, Straus SE. A pragmatic study exploring the prevention of delirium among hospitalized older hip fracture patients: Applying evidence to routine clinical practice using clinical decision support. *Implementation Science*. 2010 Oct 22;5(1):81.
